# A systematic review of 4D magnetic resonance imaging techniques for abdominal radiotherapy treatment planning

**DOI:** 10.1016/j.phro.2024.100604

**Published:** 2024-07-04

**Authors:** Lamyaa Aljaafari, David Bird, David L. Buckley, Bashar Al-Qaisieh, Richard Speight

**Affiliations:** a Leeds Institute of Cardiovascular & Metabolic Medicine (LICAMM), University of Leeds, Woodhouse, Leeds, LS2 9JT, United Kingdom; b Department of Medical Physics and Engineering, Leeds Teaching Hospitals NHS Trust, Leeds, LS9 7TF, United Kingdom; c King Saud bin Abdulaziz University for Health Sciences, Department of Diagnostic Radiology Faculty of Applied Medical Sciences, Alahssa, Saudi Arabia

**Keywords:** Four-dimensional computed tomography, Four-dimensional magnetic resonance imaging, Motion management, Abdominal radiotherapy treatment planning

## Abstract

**Background and purpose:**

Four-dimensional magnetic resonance imaging (4DMRI) has gained interest as an alternative to the current standard for motion management four-dimensional tomography (4DCT) in abdominal radiotherapy treatment planning (RTP). This review aims to assess the 4DMRI literature in abdomen, focusing on technical considerations and the validity of using 4DMRI for patients within radiotherapy protocols.

**Materials and methods:**

The review followed the Preferred Reporting Items for Systematic Reviews and Meta-Analyses (PRISMA) guidelines. A comprehensive search was performed across the Medline, Embase, Scopus, and Web of Science databases, covering all years up to December 31, 2023. The studies were grouped into two categories: 4DMRI reconstructed from 3DMRI acquisition; and 4DMRI reconstructed from multi-slice 2DMRI acquisition.

**Results:**

A total of 39 studies met the inclusion criteria and were analysed to provide key findings. Key findings were 4DMRI had the potential to improve abdominal RTP for patients by providing accurate tumour definition and motion assessment compared to 4DCT. 4DMRI reconstructed from 3DMRI acquisition showed promise as a feasible approach for motion management in abdominal RTP regarding spatial resolution. Currently,the slice thickness achieved on 4DMRI reconstructed from multi-slice 2DMRI acquisitions was unsuitable for clinical purposes. Lastly, the current barriers for clinical implementation of 4DMRI were the limited availability of validated commercial solutions and the lack of larger cohort comparative studies to 4DCT for target delineation and plan optimisation.

**Conclusion:**

4DMRI showed potential improvements in abdominal RTP, but standards and guidelines for the use of 4DMRI in radiotherapy were required to demonstrate clinical benefits.

## 1 Introduction

Radiotherapy aims to deliver the prescribed dose to the target area whilst minimising radiation to healthy tissues. For accurate treatment planning, advanced imaging techniques are used to identify the targets and organs at risk (OAR), calculate the dose, and verify positioning before and during treatment. However, there can be geometric uncertainties caused by motion which may result in underdosing the target or overdosing OAR. For abdominal radiotherapy, breathing motion is a source of uncertainty and must be managed appropriately [1]. Currently, abdominal radiotherapy relies on four-dimensional CT (4DCT) for managing motion [2]. 4DCT acquires data over several minutes over multiple breath-holds while the patient slowly moves through the CT scanner with projections being acquired and linked to when in a breathing cycle they were acquired. Data is binned based on amplitude or phase within the breathing cycle, with all projection data within that bin being used to reconstruct an image corresponding to that bin. This 4DCT data provides information about tumour motion during breathing to help with target delineation. However, 4DCT has limitations such as poor soft tissue contrast, motion artifacts, reliance on liver motion to estimate tumour movement that lead to uncertainties related to tumour position [3], [4].

As an alternative to 4DCT, four-dimensional magnetic resonance imaging (4DMRI) has emerged as a tool for planning radiotherapy. It offers better visualisation of tumours and potentially more accurate motion estimation as it does not rely on the diaphragm motion as a surrogate for tumour motion. Recently, there has been a growing interest in developing 4D MRI for radiotherapy [5], [6], [7]. These applications can broadly be categorised into multislice 2DMRI data acquisition and 3DMRI data acquisition acquired repeatedly, where the 4th dimension can be reconstructed into respiratory-correlated 4DMRI (RC-4DMRI) or time-resolved 4DMRI (TR-4DMRI). RC-4DMRI acquired over several minutes capturing breathing cycles during scanning. This is achieved by binning and averaging data acquired in multiple breathing cycles to represent the motion in different phases, hence they assume breathing is periodic. Whereas TR-4DMRI involves capturing a series of fast 3D images over time. This eliminates the need to assume periodic respiratory motion and avoids inconsistency in binning the data during 4D reconstruction.

Despite these efforts, the clinical implementation of 4DMRI in practice remains limited. To the best of the author's knowledge, there was no systematic review specifically focused on clinical requirements to develop 4DMRI in abdominal radiotherapy. Existing reviews primarily discussed different approaches and challenges in acquiring and using 4DMRI for radiotherapy [5], [6], [7]. Thus, to facilitate future research, it is essential to develop 4DMRI technologies that align with the standard requirements for MRI use in radiotherapy. The aim of this review was to evaluate the current literature reports on the use of 4DMRI during simulation in abdominal planning. Its scope focused on technical considerations and the validity of using 4DMRI for patients within radiotherapy protocols.

## 2 Method and materials

A systematic review was conducted to evaluate publications that investigated the use of 4DMRI techniques for radiotherapy treatment planning in the abdomen. The review followed the Preferred Reporting Items for Systematic Reviews and Meta-Analyses guidelines [8]. The search was performed on the Embase, Scopus, and Web of Science databases for all years up to December 31, 2023, using the search protocols in supplementary material A. A wide search criterion was implemented to ensure the inclusion of all relevant papers in the review. Articles were included that referred to '4DMRI' and 'respiratory motion' and 'radiotherapy' or their synonyms in the title and abstract. The search results for each database were combined, and duplicates were removed. The remaining results were screened using 3 levels. Primary screening involved evaluating titles and abstracts for specific use of 4DMRI in radiotherapy. Articles that focused on organs unrelated to respiratory motion or were not relevant to 4DMRI and radiotherapy were excluded. Abstracts, and review articles were excluded. The secondary screening evaluated titles and abstracts and focused on the utilization of 4DMRI in abdominal treatment planning applications. Articles related to 4DMRI acquired for online magnetic resonance guided radiotherapy (MRgRT) were excluded from the review, including studies involving tumour tracking, adaptation. Additionally, studies that used orthogonal 2D cine, 4D synthetic CT (4D sCT), solely in lung patients or involved animals were also excluded. Tertiary screening involved full-text screening, and studies acquired in volunteers or phantoms only were excluded as they did not meet the inclusion criteria for abdominal radiotherapy. The primary organs of interest in this review were the liver, pancreas, and kidneys. After identifying the eligible studies, a backward and forward citation search was conducted to identify any additional relevant studies.

The eligible studies were then categorised based on the method of data acquisition (1) 3DMRI data acquisition and (2) multi-slice 2DMRI data acquisition. Key findings from each study were summarised in data tables for each category. To be applicable in clinical settings, the included articles have been evaluated based on criteria essential for using MRI in radiotherapy [9]. These criteria include spatial resolution, slice thickness, temporal resolution, geometric fidelity of 4D MRI, field of view (FOV), scan time, reconstruction time, and methods of validation in cancer patients.

## 3 Results

### 3.1 Study selection

**Fig. 1:**
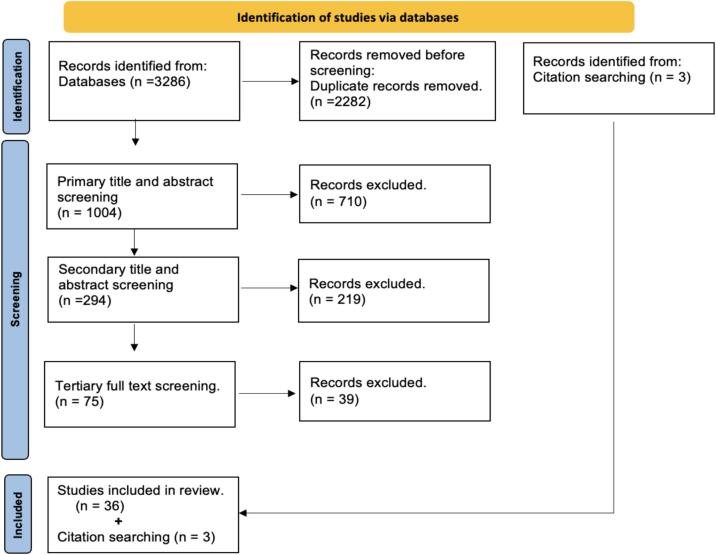
Flowchart of the systematic review process, including the number of studies included in this review.

Fig. 1 presents the results of the database search. Initially, the combined databases search produced 3286 records, which were then refined to 1004 unique records by removing duplicates. Following primary title and abstract screening, 294 records remained. Secondary screening identified 75 studies for retrieval, and out of these, 75 studies underwent tertiary full-text screening. A total of 36 studies met the inclusion criteria. Furthermore, an additional three studies were discovered through forward and backward citation searches, bringing the total number of included studies in this systematic review to 39. Table 1 provides a breakdown of the excluded articles based on their classifications and quantities.

**Table 1:** Categories and number of articles excluded from this review after primary, secondary, and full-text screening. CT: computed tomography; MRI: magnetic resonance imaging; 4D: four dimensional; 2D: two dimensional; MRgRT: magnetic resonance guided radiotherapy, sCT: synthetic CT.

Reasons for exclusion	No. of articles
Primary title and abstract screening	
Not related to 4DMRI	281
Not related to radiotherapy	103
Not related to respiratory motion	237
Review papers	74
Abstract	15
Total	710
Secondary title and abstract screening	
MRgRT (on board) application	89
Orthogonal 2D cine	60
4D sCT	27
Animals	2
4DMRI in Lung patients	41
Total	219
Tertiary full-text screening	
Abdomen 4DMRI in volunteers or phantom only	39
Total	39

### 3.2 4DMRI reconstructed from 3DMRI data acquisition

The systematic review identified 26 articles investigating 4DMRI reconstructed from 3DMRI data acquisition for abdominal radiotherapy [10], [11], [12], [13], [14], [15], [16], [17], [18], [19], [20], [21], [22], [23], [24], [25], [26], [27], [28], [29], [30], [31], [32], [33], [34], [35]. An overview of 4DMRI techniques and imaging parameters used in these studies can be found in supplementary material B, along with the key findings insupplementary material C. The studies achieved various 3D spatial resolutions, with anisotropic voxel sizes ranging from 1.1 x 1.1 x 3 mm^3^ to 3 x 3 x 5 mm^3^ [10], [13], [16], [18], [22], [23], [25], [26], [27], [28], [29], [30], [32], [35], and isotropic voxel sizes ranging from 1.56 mm^3^ to 3 mm^3^ [11], [12], [14], [15], [17], [19], [21], [24], [31], [33], [34], with scan time ranging between 49.6 s to 9 min. Additionally, a few studies have reported the temporal resolution from RC-4DMRI, with values ranging from 300 ms to 500 ms [11], [12], [14], [19] with similar results reported in TR-4DMRI [25], [28], [30], [32], [33], [34], [36]. Reconstruction time for 4DMRI techniques varied from 3 s to 10 h [10], [19], [21], [24], [25], [26], [28], [32], [33], [34]. The patient cohort size reported ranged from 1 to 43 patients with different type of abdominal cancers (liver, pancreas, kidneys, and adrenal gland) [10], [11], [12], [13], [14], [15], [16], [17], [18], [19], [20], [21], [22], [23], [24], [25], [26], [27], [28], [29], [30], [31], [32]. Eight studies [12], [18], [19], [25], [26], [27], [28], [29] included a minimum sample size of 10 patients or more, while three studies [31], [34], [35] used a sample size exceeding 20 patients.

This review highlighted 13 studies that developed RC-4DMRI reconstruction, using self-gating as a motion surrogate technique for motion detection [11], [12], [13], [14], [15], [17], [18], [19], [20], [22], [24], [28], [29]. Self-gating is solely an MRI-based approach, which relies on the MRI signal itself to quantify respiratory motion. Feng et al [13] developed a novel image reconstruction called (XD-GRASP) eXtra-Dimensional golden-angle radial MRI using compressed sensing. Five studies [14], [15], [19], [20], [24] investigated techniques aimed at improving the overall image quality of 4DMRI to achieve more accurate tumour delineation while preserving motion information. Other studies investigated different types of motion surrogates. Stemkens et al [10] used a 1DMRI navigator, while Navest et al [23] employed a noise navigator.

Limited studies have validated the accuracy of 4DMRI in comparison with 4DCT, as shown in supplementary material B [12], [18], [29]. Yang et al [12] showed the cross corelation (CC) in tumour motion was 0.91, with mean absolute difference (MAD) of 1.1 ± 0.4 mm in superior inferior (SI) direction. While the mean of standard deviation of absolute gross tumour volume (GTV) calculated from 10 breathing phases was a statistically significant reduction in 4DMRI. Oar et al [18] found the median difference between 4DCT and 4DMRI in tumour amplitude motion was 0.6 mm greater in 4DMRI. The only study that investigated dosimetric consequences was Thomas et al [29] who found statistical significant lower dosimetric coverage of planning target volume (PTV) obtained from 4DMRI compared to 4DCT when using a CT optimised plan. Specifically, the volume of the PTV receiving 90 % of the prescribed dose was 76 % in MR and 89 % in CT (p = 0.002).

Other studies have validated 4DMRI using real-time 2DMRI [11], [12], [22], the results showed CC in tumour motion trajectories, with an approximate CC value of 0.93, with mean absolute differences (MAD) of ≤ 1 mm in the SI direction [11], [12], and GTV motion was 10 % less in 4DMRI [22]. In addition, four studies [11], [12], [18], [22] demonstrated the motion accuracy of 4DMRI against phantom studies.

Five studies investigated 3D real-time TR-4DMRI reconstruction by using motion models and deep learning (DL), aiming for enhancing 3D real-time quality while minimising acquisition and reconstruction times. Romaguera et al [27] developed a deep probabilistic motion model that predict volumetric images with a mean landmark error of 1.7 ± 1.7 mm in tested MRI data. Xiao et al [31] used a DL model to developed ultra-quality (UQ) 4DMRI reconstruction, with relative motion errors less than 1 mm in all directions. Liu et al [33] investigated prior-augmented implicit neural representation learning (NeRP model), achieved similar structure similarity index (SSIM) value of 0.98 and approximately less than 2.5 mm using tumour Hausdorff distance. Xiao et al [34] used downsampling-Invariant deformable registration (D2R model) to reconstructed high quality 4DMRI with relative region of interest (ROI) motion errors less than 2.7 mm to original 4DMRI. Lastly, Stemkens et al [16] demonstrated that intrafraction variation with the principal component analysis (PCA) model could lead to GTV underdosing and was more accurate, with an root mean square error (RMSE) of 1.09, outperforming other motion models.

Other methods directly obtain real-time 3DMRI by matching motion states learned offline with motion signatures acquired in real time (MRSIGMA) [25]. This method improved image quality of liver tumours and showed positive linear correlation of (R^2^ = 0.94) compared to real-time 2DMRI. A similar technique called Live-view 4D GRASP MRI was demonstrated by Feng [32]. Another method acquired pseudo-3D real-time images through simultaneous 2D cine imaging in both sagittal and coronal planes, which were then reconstructed into isotropic resolution 4DMRI using a super-resolution technique [21]. Feng et al [25] and Mickevicius and Paulson [21] demonstrated the motion accuracy of 4DMRI against phantom studies.

Two studies developed DL 4DMRI reconstructions, Freedman et al [26] proposed deep radial convolutional neural network (4D-Dracula), which showed mean structural similarity index (SSIM) value of 0.9 and statistically insignificant median tumour motion differences of less than 2.4 mm compared to state-of-art 4D reconstruction. Murray et al,[35] developed deep space–time-coil reconstruction network without k-space data consistency (Movienet), which showed similar multiscale-SSIM and mean square errors compared to XD-GRASP reconstruction.

### 3.3 4DMRI reconstructed from multislice 2DMRI data acquisition

The systematic review identified 13 articles investigating multi-slice 2DMRI data acquisition reconstructed to 4DMRI [36], [37], [38], [39], [40], [41], [42], [43], [44], [45], [46], [47], [48]. An overview of 4DMRI techniques and imaging parameters used in these studies can be found supplementary material D, and along with the key findings (supplementary material E). Through-plane spatial resolution ranged from 4-5 mm, with in-plane pixel sizes ranging from 0.78-1.9 mm^2^. The acquisition time for the 4DMRI data ranged from 1.2 to 20 min [36], [39], [40], [41], [42], [45]. Few studies have reported the temporal resolution and reconstruction time, with temporal resolution ranging from 180 ms to 333 ms [37], [40], [41], [44], [47]. Several methods for internal motion surrogates have been reported, mutual information (MI) [40], clustering method [44], body area (BA) [37], [38], [47], 1D navigator [36], [41], [42], [45], while one study reported external motion surrogate [39]. Meschini et al [44] reported varying reconstruction times based on respiratory surrogate methods; the MI method took 2 min [40], [44], while the k-clustering approach varied between 11 and 35 min [44]. The cohort size ranged from 1 to 36 patients with abdominal cancers [36], [37], [38], [40], [41], [42], [43], [44], [45], [46], [47], [48]. One study [48] included 13 patients while three studies [40], [42], [45] used a sample size exceeding 20 patients.

Limited studies have validated the accuracy of 4DMRI in comparison with 4DCT [36], [38], [45]. A study found a good agreement in mean tumour motion trajectories, with the range of CC between 0.93 and 0.98 [38]. The mean differences in GTV centroid motion were 0.7 (LR), 0.9 mm (AP), and 1.9 mm (SI) [45] and mean differences in tumour motion amplitude were 0.74 ± 0.0 mm (SI), 0.3 ± 0.1 mm (AP), and 0.2 ± 0.1 mm (RL) [38]. Other studies compared the target volume from the two modalities. Statistically significant reduction where found in GTV and internal target volume (ITV) in 4DMRI compared to 4DCT [45]. However, 4DMRI showed an increase in liver volume and in OAR (not specified) compared to 4DCT [45]. Moreover, the Dice similarity of ITV between 4DCT and 4DMRI was 92 %-95 % for the youngest patients with similar breathing characteristics and reduced (82 %-88 %) for other patients due to variation in breathing characteristics [36]. Uh et al [36], [41] demonstrated the accuracy of 4DMRI in phantom studies.

Other studies have validated 4DMRI using real-time 2D [37], [38], [41], [48]. The CC ranged from 0.98 to 0.99, with mean differences in motion amplitude between less than 1 mm [38], and MAD in tumour motion magnitude in all direction ranged from 1.1 to 2.1 mm [37]. The MAD in diaphragm motion magnitude was found to be smaller using a diaphragm navigator compared to 2DMRI, which ranged from 0.7 to 1.8 mm in AP direction [41]. Respiratory correlated MRI fingerprinting (MRF) was assessed as a method of 4DMRI, with mean difference in motion between 2DMRI and 4DMRF of 1.5 ± 1.1 mm SI and 0.8 ± 0.6 mm AP, with mean Pearson correlation coefficient (PCC) 0.95 ± 0.05SI and 0.93 ± 0.09 AP[48]. Five studies [37], [39], [43], [44], [47] demonstrated the motion accuracy of 4DMRI against phantom studies.

## 4 Discussion

This systematic review evaluates the current literature on 4DMRI used in patients undergoing abdominal radiotherapy. It highlighted the potential benefits of 4DMRI in the planning stage. The key findings drawn from this systematic review were as follows: (1) 4DMRI has the potential to improve abdominal radiotherapy treatment planning for patients by providing more accurate tumour definition and motion assessment compared to 4DCT. However, it would be recommended to validate 4DMRI using both 4DCT and phantoms for a robust and comprehensive assessment. (2) 4DMRI reconstructed from 3DMRI acquisition shows promise as a feasible approach for motion management in abdominal radiotherapy planning in term of spatial resolution. (3) Large slice thickness currently achieved on 4DMRI reconstructed from multi-slice 2D acquisition are unsuitable for clinical purposes. (4) The current barriers for clinical implementation of 4DMRI in radiotherapy are the limited availability of validated commercial solutions and the lack of larger patient cohort studies that directly compare it to 4DCT. These comparisons are essential to highlight the potential clinical benefits of 4DMRI, particularly concerning target volume delineation and radiotherapy plan optimisation.

Several studies developed 4DMRI reconstructed from 3D acquisition that met some technical requirements for use in radiotherapy protocols. The recommended MRI image parameters for clinical implementation in radiotherapy require a spatial resolution of ≤ 1 mm and slice thickness of < 3 mm [9]. However, acquiring a 1 mm spatial resolution in MRI results in long scan times from a clinical standpoint. Scan time remains a challenge in most 4DMRI techniques. Therefore, there currently is a trade-off between spatiotemporal resolution and scan time due to MRI scanner constraints. High spatial resolutions of approximately 1x1x3 mm^3^ have been achieved [13], [18], [23], [26], [29] with scan time varies from less than 2 min to 10 min. However, Feng et al [13] and Oar et al [18], Freedman et al [26], who were able to obtain short scan time of around 5 min or less with high spatial resolution. Another technical consideration is the choice of the number of bins, which varies with each method. Increasing the number of bins to match the 10 used in 4DCT could potentially increase both the reconstruction burden and the issues related to undersampling.

Meanwhile, 4D image reconstruction times have not been thoroughly reported. 4D reconstruction requires processing large amount of data, complex reconstruction algorithms and adequate hardware such as processing power, memory, and storage. All of these can be optimised to reduce reconstruction times to a clinically acceptable level. Ideally, near real time would be acceptable clinically because it is important to ensure the quality of the 4DMRI before the patient leaves the scanner or hospital. If any artifacts or errors are detected, it is more efficient to repeat the scan immediately rather than calling the patient back for a repeat scan later. This will ensure the best patient experience and enhances the efficiency of the radiotherapy department. Future developments in MRI technology could get around these constraints by reducing scan and reconstruction times using cutting-edge methods such compressed sensing [13], [49], [50], SMS [21], [51], or DL reconstruction [26], [33], [34], [35], [52]. The application of DL reconstruction has shown promise with a reconstruction time of around 3 s [34], 28 s [26], and 0.73 s [35]. However, DL methods are still in the early stages of validation, and this need further investigation to be used in clinical settings [26], [33], [34], [35].

Several studies attempted to reconstructed 4DMRI from multislice 2DMRI acquisition using retrospective or prospective sorting, which involves acquiring multiple 2DMRI slices to reconstruct 3D volumes, and then binning them into respiratory phases or amplitude bins. Different internal motion surrogates have been proposed [36], [37], [38], [40], [41], [42], [43], [44], [45], [46], [47] and the advantages of using internal motion surrogates over external motion surrogates have been demonstrated [39]. These advantages include simplified equipment setup, enhanced image quality, improved synchronization with image acquisition, and a more reliable representation of internal structures. Regardless of the motion surrogates used [36], [37], [38], [40], [41], [42], [43], [44], [45], [46], [47], [48], all of the reported methods have obtained a relatively large slice thickness of around 5 mm. This slice thickness is inadequate for accurate treatment planning purposes, which require < 3 mm [9]. Therefore, it is crucial to consider optimising the acquisition parameters to achieve thinner slice thickness with no slice gap. Another consideration is that high spatial and temporal resolutions require a longer scan time to collect sufficient data for reconstruction. Significant reduction in scan time have been achieved by Chen et al [45] with 5 min scan times using a 1D navigator, while the MI method allowed similar image quality with a reduced scan acquisition time of 1.2 min [40].

For both 3D or multislice 2DMRI acquisitions used to reconstruct 4DMRI, the common image contrast is typically obtained from T2/T1w GRE and T1w GRE sequences. This choice is primarily made to achieve high spatiotemporal resolution. However, it is important to note that T2w 4DMRI has been explored in multislice 2DMRI sequences [36], [39], [43], [45]. Nevertheless, it still faces challenges for clinical implementation, particularly due to the relatively large slice thickness of 5 mm. On the other hand, employing T2 3DMRI sequences requires a longer acquisition time and remains a challenge in the context of 4DMRI.

Another important technical concern in using MRI for radiotherapy is geometric distortion. This review revealed that there is a lack of research investigating the potential geometric distortions in abdominal 4DMRI techniques. Geometric distortion can compromise the spatial accuracy of MRI, which is particularly crucial for tumour localisation in radiotherapy. Solutions for reducing geometric distortion such as post processing geometric distortion correction algorithms are essential [9]. For most radiotherapy applications, a residual distortion errors of 2 mm considered acceptable. The 4D phantom study demonstrated geometric accuracy up to 1.31 mm [53], another study reported maximum of 2.5 mm error [54]. Therefore, future studies should test the reliability of geometric distortions in 4DMRI acquisition methods in patients and develop strategies for addressing potential distortions in order to use these methods for clinical use in radiotherapy planning.

There are several ways to validate 4DMRI, each with its own limitation. Firstly, phantoms are more controlled with predefined motion, allowing for a direct comparison between the expected and measured movement. However, phantoms simplify the complexity and variability of human anatomy and physiology as well as not precisely replicating the characteristics of human tissues and thus results may not be directly translatable to patient results. Secondly, real-time 2DMRI provides immediate imaging of motion on the same MR scan position and time, however it might not represent the full 3D motion. Lastly, 4DCT is commonly used in clinical practice for evaluating respiratory motion in radiotherapy, but it lacks contrast for abdominal tumours, often making them invisible which limits the usefulness of comparison in the region of the tumour. Additionally, external motion surrogates don't accurately match the actual motion of tumours and internal organs. Therefore, combining multiple validation methods to overcome individual limitations is desirable to achieve a comprehensive motion accuracy assessment. Several studies have validated motion accuracy of 4DMRI techniques in patients against 4DCT and phantoms [12], [18] and real-time 2DMRI and phantoms [10], [12], [22], [25], [37]. Yet, a lack of validation against 4DCT and phantoms is notable. Whereas a few studies validated 4DMRI motion accuracy in patients against either 4DCT [36], [45] or real-time 2DMRI [38], [41], [48], However, it would be recommended to ensure a robust and comprehensive validation of 4DMRI using both 4DCT and phantoms. Firstly, phantoms allow direct, accurate, and reusable measurement comparisons, which confirm that all technological components of the 4DMRI are working optimally. Secondly, 4DCT has been widely accepted as a clinical standard to evaluate respiratory motion, thus, a reliable benchmark is provided by comparing 4DMRI to 4DCT in patient data. It is beneficial to understand how 4DMRI relates to what is currently accepted. By integrating both in the validation process, researchers can ensure both technical precision and clinical relevance of the results, which ultimately leads to increased levels of confidence in the use of 4DMRI in clinical settings.

Regardless of the 4DMRI reconstruction methods using 3D or multislice 2DMRI acquisitions, 4DMRI motion accuracy showed high agreement in tumour motion pattern compared to 4DCT and real-time 2DMRI, especially in the SI direction, however moderate correlation in the other direction. This could be due to the hysteresis effect. Several studies have demonstrated the presence of hysteresis in motion using 3D motion model trajectories [10], [11], [16], [27]. However, this hysteresis effect is often underestimated when using respiratory belts in 4DCT, as the signal does not accurately represent the internal motion of the tumour [17]. On the other hand, 2DMRI captures motion in only one direction, which limits the understanding of hysteresis motion. Thomas et al [29] demonstrated that the two-directional binning method revealed an additional displacement of at least 3 mm of anterior motion, due to hysteresis effects. Therefore, 4DMRI has a greater potential for reliable motion evaluation, owing to its unique feature of using internal motion surrogates, which can enhance the accuracy in tracking tumour motion.

A small number of studies reported differences in target volumes between 4DMRI and 4DCT. 4DMRI showed a reduced GTV and ITV compared to 4DCT [36], [38], [45] which is hypothesised to be due to MRI's superior soft tissue contrast compared to CT. Traditional radiation treatment planning uses 4DCT scans to estimate GTV-ITV margins for abdominal patients, but this method is over-conservative as tumours are not always visible on CT so structures like the liver wall that can be visualised in both images must be used as a surrogate. This leads to uncertainty and consequently, oncologists use larger margins to avoid under-treatment of targets, which exposes healthy tissue to higher radiation doses than necessary. 4DMRI, by contrast, provides clear visualisation of tumours and their motion, allowing for more accurate and precise ITV definition. This is hypothesised to result in smaller treatment volumes, minimising radiation to healthy tissue and potentially reducing patient toxicity due to the treatment. A single study conducted a comparative assessment of target volumes and plan dosimetry optimisation between 4DCT and 4DMRI [29]. The study highlighted that when optimising treatment plans based on PTVs derived from 4DCT there was a statistically significant lower dosimetric coverage of PTVs derived on MRI. Therefore, the failure to incorporate PTVs derived from 4DMRI in the planning process may lead to suboptimal dosing of the actual PTV. However, it is crucial to validate 4DMRI before use in order to accurately capture both motion and volume, hence enhancing the accuracy of treatment planning. 4DMRI has the potential to enhance the coverage of prescribed radiation doses to target volumes, while minimising the radiation exposure to surrounding healthy tissues. Yet, drawing a definitive conclusion can be challenging when limited studies in the literature compare dosimetric assessment with 4DCT plan.

4DMRI reconstructed from multislice 2D and 3D acquisition demonstrated similar accuracy. However, their inherent differences must be taken into consideration. Although they showed similar tumour motion accuracy, their clinical advantages over 4DCT have not been thoroughly investigated. The current studies focused on the differences in motion amplitude between 4DCT and 4DMRI, instead of exploring the possible clinical benefits of 4DMRI compared to 4DCT such as tumour volume changes and treatment plan differences due to this change. 4DMRI reconstructed from 3D acquisition appears more promising for use in radiotherapy, providing acquisitions closer to the clinical specifications. However, the majority of the studies included small patient’s cohorts, which limited the generalisability. Furthermore, patient validation required further investigation using a larger cohort. It is hypothesised that larger cohort studies have not been presented so far in the literature due to the limited availability of validated commercial solutions for 4DMRI in RT from vendors, as well as the rarity of abdominal cancers, such as in the liver and pancreas, making large cohort studies more challenging.

Future work should focus on optimising the parameters of multi-slice 2DMRI to meet the specific needs of treatment planning. Furthermore, comparing dosimetric plans derived from 4DMRI and 4DCT could reveal the clinical advantages of each modality. It is also important for MRI vendors to support these advancements in 4DMRI and ensure its validation for clinical use. Currently, all these developments are institution-based as there are limited commercial solutions available for clinical implementation. To the best of our knowledge, one vendor has a product designed for 4DMRI in RT [55]. However, more studies are needed to validate this product in larger patient cohorts. Additionally, an important consideration is the need for standardisation of both quantitative and qualitative metrics among different studies. This is essential to facilitate future research to perform more comprehensive comparisons. Currently, the diverse methods and metrics used across different studies pose a challenge to drawing reliable and valid conclusions to guide 4DMRI for clinical implementation.

This review is not without limitations. Primarily, the screening was conducted by a sole reviewer, the scope of the articles was limited to papers written in English, and therefore introducing an element of selection bias. Next, the author also excluded studies involving phantoms or volunteers only. Instead, the review was exclusive to the assessment of clinical validation in actual patients. Finally, studies related to developing 4DMRI for online MRgRT were excluded since their primarily focus is on adaptation and tumor tracking. However, more centres have access to MR simulation and therefore the goal of this study was to evaluate treatment planning options rather than to guide treatments online.

4DMRI had continuously evolved and improved over the years, which may support the growing interest in implementing an MRI-only pathway for enhancing abdominal radiotherapy treatment planning. It potentially offered advantages over 4DCT, such as reduced tumour margins and sparing healthy tissue. However, standards and guidelines were needed to ensure the consistency and reliability of these clinical benefits. While 4DMRI reconstructed from 3DMRI acquisitions was feasible for clinical implementation, 4DMRI reconstructed from multislice 2DMRI was not currently clinically acceptable due to unsuitable through-plane resolution. The development of the reported techniques has focused on research applications, highlighting the need for validated commercial solutions.

## Funding

L Aljaafari’s is a lecturer funded by King Saud bin Abdulaziz University for Health Sciences, Alahssa, Saudi Arabia.

## CRediT authorship contribution statement

**Lamyaa Aljaafari:** Writing – original draft, Conceptualization, Methodology, Investigation, Visualization. **David Bird:** Writing – review & editing, Conceptualization, Methodology, Investigation, Supervision, Project administration. **David L. Buckley:** Writing – review & editing, Conceptualization, Methodology, Investigation, Supervision, Project administration. **Bashar Al-Qaisieh:** Writing – review & editing, Conceptualization, Methodology, Investigation, Supervision, Project administration. **Richard Speight:** Writing – review & editing, Conceptualization, Methodology, Investigation, Supervision, Project administration.

## Declaration of Competing Interest

The authors declare that they have no known competing financial interests or personal relationships that could have appeared to influence the work reported in this paper.
