# Supplementary material for: A systematic review of 4D magnetic resonance imaging techniques for abdominal radiotherapy treatment planning
Source: Phys Imaging Radiat Oncol. 2024 Jul 4;31:100604. doi: 10.1016/j.phro.2024.100604 (PMC11283022; doi:10.1016/j.phro.2024.100604)
Supplement: Supplementary Data 1 [file mmc1.docx]

**Supplementary material A**

Search protocol used for all four databases to obtain relevant studies for the review.

| Databases | # | Search terms |
| --- | --- | --- |
| Medline database | 1 | (4D or "4 dimen*" or "four dimen*" or cine or cinematic or dynamic).tw |
|  | 2 | Magnetic Resonance Imaging, Cine/ |
|  | 3 | MR or MRI or "Mag* res* im*").tw. |
|  | 4 | 1 and 3 |
|  | 5 | 2 or 4 |
|  | 6 | (motion or breathing cycle* or "respiratory-correlated MR" or "time-resolved MR" or gated or gating or free-breathing or moving target* or breath-hold*).tw. |
|  | 7 | Breath Holding/ |
|  | 8 | 6 or 7 |
|  | 9 | (RT or radiother* or radi* therapy or "radiation oncology").tw. |
|  | 10 | Radiotherapy/ |
|  | 11 | 9 or 10 |
|  | 12 | 1. and 8 and 11 |
| Embase  database | 1 | (4D or "4 dimen*" or "four dimen*" or cine or cinematic or dynamic).tw. |
|  | 2 | (MR or MRI or "Mag* res* im*").tw |
|  | 3 | cine magnetic resonance imaging/ |
|  | 4 | 1 and 2 |
|  | 5 | 3 or 4 |
|  | 6 | (motion or breathing cycle* or "respiratory-correlated MR" or "time-resolved MR" or gating or gated or free-breathing or moving target* or breath-hold*).tw. |
|  | 7 | breathing/ or breath holding |
|  | 8 | 6 or 7 |
|  | 9 | (RT or radiother* or radi* therapy or "radiation oncology").tw. |
|  | 10 | radiotherapy/ |
|  | 11 | 9 or 10 |
|  | 12 | 1. and 8 and 11 |
| Scopus | 1 | (TITLE-ABS-KEY (4d OR “4 dimen*” OR “four dimen*” OR cine OR cinematic OR dynamic) |
|  | 2 | TITLE-ABS-KEY (mr OR mri OR “Magnetic resonance imaging”) |
|  | 3 | TITLE-ABS-KEY (motion OR “breathing cycle*” OR “respiratory-correlated MR” OR “time-resolved MR” OR gating OR gated OR “free-breathing” OR “moving target*” OR “breath-hold*”) |
|  | 4 | TITLE-ABS-KEY (rt OR radiother* OR “radi* therapy” OR “radiation oncology”)) |
|  | 5 | 1 AND 2 AND 3 AND 4 |
| Web of Science | 1 | (4d OR “4 dimen*” OR “four dimen*” OR cine OR cinematic OR dynamic) (all field) |
|  | 2 | (mr OR mri OR “Magnetic resonance imaging”) (all field) |
|  | 3 | motion OR “breathing cycle*” OR “respiratory-correlated MR” OR “time-resolved MR” OR gating OR gated OR “free-breathing” OR “moving target*” OR “breath-hold*”) (all field) |
|  | 4 | (rt OR radiother* OR “radi* therapy” OR “radiation oncology”) (all field) |
|  | 5 | 1 AND 2 AND 3 AND 4 |

| Supplementary material B | | | | | | | | |
| --- | --- | --- | --- | --- | --- | --- | --- | --- |
| Overview of the key imaging parameters and methodologies used for different abdominal organs to reconstruct 4DMRI from 3DMRI data acquisition | | | | | | | | |
| Author  [ref] | **Organ**  **(patient number)** | **Acquisition type** | **Image contrast** | **Surrogate** | **Binning Meths** | **Spatial Resolution**  **(Temporal**  **Resolution)** | **FOV** | **Scan Time**  **(Recon. Time)** |
| Stemkens  (2015) [10] | Pancreas (6) | 3D RSOS | T2/T1w  3D GRE | 1D nav | 10 Phase | 2.0×2.0×4.0 mm^3^  (NR) | 330x330x96 mm^3^ | 8 min 35 s  (2 min 44 s) |
| Deng (2015)[11] | Liver (2) | 3D Radial | T1w  3D GRE | SG | 10 Phase | 1.56×1.56×1.56 mm^3^  RC (300-500 ms) | 300×300×300 mm^3^ | 8 min (NR) |
| Yang (2015)[12] | Pancreas (10) | 3D Radial | T1w  3D GRE | SG | 10 Phase | 1.56×1.56×1.56 mm^3^  RC (300-500 ms) | 300×300×300 mm^3^ | 8 min (5 hrs) |
| Feng  (2016)[13] | Liver (1) | 3D RSOS | T1w  3D GRE | SG | 4 Phase | 1.1×1.1×3 mm^3^  (NR) | 360×360×240 mm^3^ | ∼95 s  (∼15 min/slice) |
| Jin  (2016) [14] | Pancreas (9) | 3D Radial | T1w  3D GRE | SG | 10 Phase | 1.6x1.6x1.6 mm^3^  RC (300-500 ms) | 300x300x300 mm^3^ | 8 min (NR) |
| Deng  (2017) [15] | Liver (1)  Pancreas (3) | 3D Radial | T1w  3D GRE | SG | 10 Phase | 1.56×1.56×1.56 mm^3^  (NR) | 400x400x400 mm^3^ | 7 min 23 s (NR) |
| Stemkens  (2017)[16] | Kidney (5) | 3D RSOS | T2/T1w 3D GRE | SG | 10 Phase | 1.9×1.9×2 mm^3^  TR (360 ms) | 300–350×300 –350×132– 200 mm^3^ | 3 min 22 s –  5 min 3 s (NR) |
| Breuer (2018)[17] | Liver (2) | 3D Cartesian | T1w  3D GRE | SG | 10  Amplitude | 2.1×2.1×2.1 mm^3^  (NR) | 400×400×185 mm^3^ | 5 min  36 s (NR) |
| Oar  (2018)[18] | Liver (5)  Pancreas (3) | 3D RSOS | T1w  3D GRE | SG | 5  Amplitude | 1.2×1.2×3.0 mm^3^  (NR) | 380×380 mm^2^ | 5 min (NR) |
| Yang (2018)[19] | Pancreas (10) | 3D Radial | T1w  3D GRE | SG | 10 Amplitude | 1.56×1.56 ×1.56 mm^3^ RC (300-500 ms) | 400×400×400 mm^3^ | 5 min  (8 hrs) |
| Deng (2019)[20] | Liver (1)  Pancreas (7) | 3D RSOS | T1w  3D GRE | SG | 10 Amplitude | 1.98x1.98x1.98 mm^3^  (NR) | 380 ×380×206 mm^3^ | 9 min (NR) |
| Mickevicius and Paulson  (2019)[21] | Liver (3) | SR -SOPI | T1w 3D  GRE | 1D nav | 6 Phase | 2.5 mm^3^ to 2.9 mm^3^  (NR) | 360 x 360 mm^2^ | 6 min  30 s (80 min) |
| Stemkens  (2019)[22] | Kidney (5) | 3D RSOS | T2/T1w 3D GRE | SG | 5 Amplitude and Phase | 1.9×1.9×4.0 mm^3^  (NR) | 300×300×200 mm^3^ | 3.5-5 min (NR) |
| Navest (2020)[23] | Pancreas (1) | 3D RSOS | T1w  3D GRE | Noise nav | 7 Phase | 1.3x1.3x3 mm^3^  (NR) | 400x197x400 mm^3^ | NR |
| Weick (2020)[24] | Liver and adrenal gland (4) | 3D Cartesian | T1w  3D GRE | SG | 10  Amplitude | 2.1x2.1x2.1 mm^3^  (NR) | 400×400×185 mm^3^ | 3-5 min /  330 min |
| Feng (2021)[25] | Liver (15) | 3D RSOS | T1w  3D GRE | SG | 10 Amplitude | 1.25×1.25×5 mm^3^  1.50×1.50×4 mm^3^  TR (300ms) | 320×320 mm^2^  384×384 mm^2^ | 2 min 18s  8.5 min  (73.82 min) |
| Freedman  (2021)[26] | Liver (3)  Pancreas (17) | 3D RSOS | T1w  3D GRE | SG | 16 Phase | 1.25x1.25x3.3 mm^3^  (NR) | 400×400 mm^2^ –  480×480 mm^2^ | 4 min 21s –  5 min 45 s  (28 s) |
| Romaguera,  (2021)[27] | Liver (11) | 3D RSOS | T2/T1w 3D GRE | SG | 10 Phase | 1.5×1.5×5 mm^3^  (NR) | 450×450×250 mm^3^ | 3 min (NR) |
| Mansour  (2022)[28] | Liver (11) | 3D RSO | T2/T1w 3D GRE | SG | 10 Phase | 2×2 ×2.5 mm^3^  TR (450ms) | NR | 8 min (10 hrs) |
| Thomas (2022)[29] | Liver (12) | 3D RSOS | T1w  3D GRE | SG | 4 amplitude^1^ | 1.3x1.3x3 mm^3^  (NR) | 480 x 480 mm^2^ | 8–10 min  (NR) |
| Wong (2022)[30] | Liver (2)  Pancreas (1) | 3D Cartesian | T1w  3D GRE TWIST | rPPV | NR | 2.7x2.7x4.5 to 3x3x5 mm³  TR (333 ms) | 350-382 mm^2^ | NR |
| Xiao (2022)[31] | Liver (39) | 3D Radial | T1w 3D GRE  TWIST | BA | 10 Phase | 2.7×2.7×2.7 mm^3^  (NR) | NR | 50s  (NR) |
| Feng  (2023)[32] | Liver (7) | 3D RSOS | T2/T1w 3D GRE | 2D nav | 10 Phase | 1.4×1.4x5 mm^3^  TR (0.3s/volume) | 360 × 360 mm^2^ | 2 min  49 s (NR) |
| Liu  (2023)[33] | Liver (7) | 3D RSOS | T2/T1w 3D GRE | SG | 21 Phase | 2-2.45 x 2-2.45 x 3-4 mm^3^  TR (340 ms) | 192 x 192 mm^2^ | 5 min  (4.5 min) |
| Murray (2023)[35] | Abdominal tumours (21) | 3D RSOS | T1w  3D GRE | SG | 4 and 10 amplitudes | 1.25–1.5 x 1.25–1.5 x4–5 mm  (NR) | NA | ∼1- 2.25 min (0.69 and 0.75s) |
| Xiao (2023)[34] | Liver (43) | 3D Cartesian | T1w  3D GRE TWIST | BA^2^ | 10 Phase | 2.7 x 2.7 x 2.7 mm^3^  TR (500 ms) | 432 x 345 x 172 mm^3^ | 49.6 s (3 s) |
|  | Liver (5) | 3D Cartesian | T1w  3D GRE  THRIVE | NR | NR | 3.0 x 3.0 x 3.0 mm^3^  TR (500 ms) | 420 x 420 x 159 mm^3^ | 64 s (NR) |
| *Abbreviations*: NR: not reported, min: minutes, hrs: hours, RSOS: 3D Radial stack-of stars, nav: navigator, SG: Self-gating, SR-4D-SOPI: Super-resolution simultaneous orthogonal plane imaging, TWIS: time-resolved imaging with interleaved stochastic trajectories, rPPV: The respiratory positional probability volume, Recon: reconstruction RC: Respiratory correlated. TR: Time resolved.  ^1^ Amplitude binning: Two-directional amplitude where the data in each amplitude bin is divided into inspiration and expiration groups based on the motion direction.  ^2^ Body area (BA): an internal motion surrogate, which is using changes of body surface area to extract the breathing signal. | | | | | | | | |

| Supplementary material C | | | |  |  |
| --- | --- | --- | --- | --- | --- |
| Summary of the key findings from 4DMRI reconstructed from 3DMRI data acquisition | | | | | |
| Author (Year)[Ref] | **Validation comparing to 4DMR** | **Validation metrics** | **Other key information** | | |
|  |  |  | **Motion and volume** | | **Overall image quality of proposed 4DMRI** |
| Stemkens (2014)[10] | NA | Peak-to-Peak motion | -7.4 ± 1.6 mm duodenum.  -5.4 ± 0.7 mm tumour | | (1) MRI navigator more accurate than respiratory bellows.  (2) Radial sampling reduced undersampling and artifacts |
| Deng  (2015)[11] | real-time 2DMRI | Tumour CC | 0.938 (SI), $<$ 0.8 (AP, LR) | | (1) Less overall image quality (i.e. signal inhomogeneity) and variation in tissue contrast  (2) No statistically significant difference in image sharpness |
|  |  | MAD | $\leq$ 1.2 mm in (SI) | |  |
|  |  | MDD | $<$-0.8 mm (SI),$<$-1.6 mm (AP),$<$ -0.7 mm (LR) | |  |
| Yang  (2015)[12] | real-time 2DMRI | Tumour CC | 0.9 ± 0.0 (SI), 0.8 ± 0.1 (AP), 0.7 ± 0.18 (LR) | | (1) Free of stitching artifacts |
|  |  | MAD | 0.8 ± 0.5 mm (SI), 0.4 ± 0.2 mm (AP), 0.5 ± 0.2 mm (LR) | |  |
|  | 4DCT | Tumour CC | 0.9 ± 0.06 (SI), 0.7 ± 0.16 (AP), 0.4 ± 0.2 (LR) | |  |
|  |  | MAD | 1.1 ± 0.4 mm (SI), 0.5 ± 0.2 mm (AP), and 0.5 ± 0.3 mm (LR) | |  |
|  |  | SD-GTV from  10 phases | 0.8 1± 0.54 cm³ 4DMRI, 1.02 ± 0.67 cm³ 4DCT | |  |
| Feng (2016)[13] | XD-GRASP  vs iGRASP | NA | NA | | (1) Improved overall image quality with less motion artifact compared to iGRASP |
| Jin  (2016)[14] | de-noised vs  raw data 4DMRI | Tumour CC | 0.9994 ± 0.0003 | | (1) Improved overall image quality |
|  |  | SD-GTV from 10 phase | 0.6 ± 0.4 cm³ 4DMRI  0.8 ± 0.6 cm³ 4DCT | |  |
| Deng (2017)[15] | SS-4D-MRI vs  NS-4D-MRI | Tumour CC | 0.96 ± 0.06 (SI), 0.78 ± 0.2 4 (AP) and 0.46 ± 0.44 (RL) | | (1) Superior vessel delineation and higher SNR and CR  (2) Fewer streaking artifacts (4-point scale) |
| Stemkens (2017)[16] | Motion models^1^ vs  real-time 2DMRI | mean RMSEs | 2.69 ± 0.68 mm STATIC, 2.37 ± 0.78 mm AVG-RESP ,1.09 ± 0.47 mm PCA model | | NA |
| Breuer (2018)[17] | 4DCT | NA | NA | | (1) Superior lesion visualisation compared to 4DCT |
| Oar  (2018)[18] | 4DCT | Median Tumour ED | 11.2 mm 4DCT,10.1 mm 4DMRI  median difference: -0.6 mm | | (1) Superior edge detection and over all image quality (four-point scale) |
| Yang (2018)[19] | SS-4D-MRI vs  NS-4D-MRI | CC | 0.93 ± 0.10 (SI), 0.65 ± 0.31 (AP), 0.77 ± 0.23 (LR) | | (1) Enhanced imaging signal and increased vessel CNR compared to NS-4DMRI and 4DCT |
| Deng (2019)[20] | MoCoAve^2^ vs  non- MoCoAve | Tumour CC | 0.91 ± 0.08 (SI), 0.51 ± 0.44 (AP), 0.37 ± 0.23 (LR) | | (1) Higher SNR, fewer artifacts and increased in overall image quality  (3-point scale) (2) No statistically significant difference in image sharpness |
|  |  | Tumour MAD | 0.5 ± 0.3 mm (SI) | |  |
|  |  | SD-GTV from 10 phases | 1.4 8±1.35 cm³ MoCoAve, 2.17±1.31 cm³ non- MoCoAve | |  |
| Mickevicius and Paulson (2019)[21] | SR reconstructions | NA | NA | | (1) The respiratory motion was well-defined without any mismatching of sorted slices |
| Stemkens (2019)[22] | 5DMRI^3^, CE-4DMRI vs real-time 2DMRI | SI GTV motion | CE-4DMRI demonstrated approximately 10% smaller motion than real-time 2DMRI | | (1) 5DMRI reduced the motion artifact compared to DCE |
| Navest (2020)[23] | Noise-nav vs SG | NA | NA | | (1) Less streaking artifacts compared to self-gating |
| Weick (2020)[24] | Dir-Reg^4^ vs  nn-Reg IR^4^ | Median COV all ROIs | Reduced by 5.6% ± 5.3% for exhalation and 7.0% ± 4.2% for inhalation compared to the nn-Reg | | (1) Superior overall image quality compared to nn-Reg (4-point scale) |
| Feng (2021)[25] | real-time 2DMRI | R^2^ | 0.948 | | (1) Suspected lesions were clearly visible in the 3D images across three planes. |
| Freedman (2021)[26] | 4D-Dracula^5^ vs  4D-MoCo^5^ | Median tumour ranges | Below 2.4 mm. | | (1) Lower overall image quality (2) Clinically acceptable for contouring in RT (four-point scale) |
|  |  | Median inter-observer DSC | $>$0.86 GTV | |  |
| Romaguera (2021)[27] | 4DMRI vs  Ultrasound | Volumetric Prediction Mean Error | 1.67 ± 1.68 mm | | NA |
| Mansour (2022)[28] | XD-GRASP and DR | NA | NA | | (1) The liver motion clearly showed the positions of both the tumour and liver in 10 phases |
| Thomas (2022)[29] | 4DCT | Dosimetric Coverage of PTVs from CT and MRI plans | - No significant difference when optimised on their respective target - Statistically significant lower of MRI target compared to CT target when they optimised on CT plan | | (1) Equal or better liver tumours clarity at extreme phases (4-point Likert scale)  (2) Higher sharpness scores at extreme phases |
|  |  | Mean liver dose, OAR and ITVs volume | No statistically significant difference between CT and MR optimised plans | |  |
| Wong  (2022)[30] | 4DCT | ITV volume | The ITV derived from 4DCT was statistically insignificantly larger than PPV144,0% and rPPV144,5% | | NA |
| Xiao  (2022)[31] | UQ 4DMRI generated by DDEM | Tumour motion errors (mm) | <1 mm in all directions | | (1) Significantly enhanced overall image quality |
| Feng  (2023)[32] | 2D nav or 1D nav vs SSIM curve^6^ | Motion CC | 0.886 ± 0.049 2D nav, 0.791 ± 0.148 1D nav | | (1) The 2D nav outperformed the 1D nav in detecting respiratory variations and/or body movements during the off-view stage |
| Liu  (2023)[33] | NeRP model^7^ | SSIM | 0.98 ± 0.01 | | (1) Image quality deceased at outside the two training phases. |
|  |  | GTV HD-95% | 2.41 ± 0.77 mm | |  |
|  |  | Mean GTV motion | <1 mm in all directions. | |  |
| Murray (2023)[35] | Movienet^8^  Vs XD-GRASP | MS-SSIM | 0.93–0.96 Movienet, 0.98–0.99 XD-GRASP | | (1) Significantly removed streaking artifact and improve overall image quality |
|  |  | MSE | $<$0.02 Movienet, $<$0.0005 XD-GRASP: | |  |
| Xiao (2023)[34] | HQ Mp D2R model^9^ vs LQ 4DMRI | Relative ROI ME | <2.7 mm in all directions. | | (1) Significantly enhanced image quality |
| *Abbreviations:* CC: Correlation Coefficient, CE-4DMRI: Contrast-enhanced 4DMRI, COV: Coefficient of variation, CR: contrast resolution, DCE: dynamic contrast enhancement, DDEM: dual-supervised deformation estimation model, dir-Reg: direct image registration, DR: Deformable registrations, DSC: Dice coefficient, ED: Euclidean Distances, SR: Super-resolution, HD: Hausdorff distance, iGRASP: iterative Golden-angle Radial Sparse Parallel MRI, MAD: Mean Absolute Difference, MDD: Motion Displacement Difference, ME: Motion errors, MoCo: 4D joint motion-compensated high-dimensional total variation algorithm, MoCoAve: motion correction averaging, MS-SSIM: multiscale- structure similarity index.NA: not applicable, Nav: navigator, nn-Reg: non-direct image registration, NS-4DMRI: non-selective excitation 4DMRI, rPPV: respiratory positional probability volume, RMSE: root means square error, ROI: region of interest, SD: Standard deviation, SG: Self-gating surrogates, SNR: signal to noise ratio, SR: Superior Reconstruction, SS-4DMRI: slab selective excitation 4DMRI, SSIM: structure similarity index, UQ: Ultra-quality, and XD-GRASP: Xtra-Dimensional Golden-angle Radial Sparse Parallel MR.  ^1^ Motion models: STATIC model, which assumes no motion and uses a mid-position 3D reference for treatment planning; AVG-RESP model, which determines respiratory phase using real-time 2DMRI, producing a time-weighted 4DMRI to reflect individual respiratory phases during radiation, and PCA model uses principal component analysis on 4DMRI deformation vector fields to generate a scalable motion model, adjusting for drifts and variations in breathing patterns by corresponding to real-time 2DMRI.  ^2^ MoCoAve: post-processing technique.  ^3^ 5DMRI: 4DMRI at different timepoints post contrast  ^4^ Dir-Reg: non-rigid image registration where all the phases directly register to end-exhale and end-inhale, and nn-Reg: non-rigid image registration where consecutive neighbouring phases are registered until the end-exhale and end-inhale in reached.  ^5^ D-Dracula 4D image reconstruction uses deep radial convolutional neural network, 4D-MoCo: 4D image reconstruction called 4D joint motion-compensated high-dimensional total variation,  ^6^ SSIM curve: The validation method involved calculating the SSIM between the most recent 3D image and all previous 3D images for each patient, resulting in a signal curve considered a ground-truth (3D reference).  ^7^ NeRP model: is an advanced MRI technique that uses sparse sampling and neural representation learning with a sparse prior for efficient 3D motion tracking in real-time.  ^8^ Movienet: a deep learning approach for 4D-MRI reconstruction  ^9^ D2R model uses a downsampling-invariant deformable registration (D2R) model for real-time, multi-parametric 4D image reconstruction. | | | | | |

| Supplementary material D | | | | | | | | |  |
| --- | --- | --- | --- | --- | --- | --- | --- | --- | --- |
| Overview of the key imaging parameters and methodologies used for different abdominal organs to reconstruct 4DMRI from multislice-2DMRI data acquisition | | | | | | | | |  |
| Author  (year)[Ref] | **Organ**  **(Patients number)** | **Acquisition type** | **Sequence**  **(Image contrast)** | **Surrogate** | **Binning**  **Methods** | **Spatial Resolution**  pixel size, slice thickness  **(Temporal Resolution)** | **FOV** | **Scan**  **Time**  **(Recon. Time)** | |
| Liu  (2014) [37] | Liver (7) | 2D Cartesian, cine, sagittal and axial | T2/T1w  GRE | BA | 10 Phase | 1.9×1.9 x5 mm  (333 ms) | 480×480mm | NR | |
| Yang (2014)[38] | Liver (7) | 2D Cartesian, cine, axial | T2/T1w  GRE | BA | Phase | 1.6–2.5×2.8–3.75  x5 mm (333 ms) | 300–480×  360–480 mm | NR | |
| Glide-hurst (2015)[39] | Stomach (1)  Liver (1) | 2D Sagittal | T2w-TSE | Respiratory bellows | 4,8 Amplitude | 1×1x4 mm (NR) | 380×210x200 mm | 7–11 min (NR) | |
| Paganelli  (2015)[40] | Liver (24) | 2D Cartesian, interleaved, Oblique | T2/T1w  GRE | MI | 8 Amplitude and Phase | 1.28 ×1.28x5 mm  (180 ms) | 320×285x100 mm | 1.2 min (2 min) | |
| Uh  (2016) [41] | Abdominal tumours (6) | 2D Cartesian,  cine, coronal | T2/T1  GRE | Diaphragm nav | 10 Amplitude probability | 1.8×1.8 x5 mm  (333 ms) | 350×350x100 mm | 8 min 12 s (NR) | |
| Uh  (2017)[42] | Abdominal tumours (36) | 2D Cartesian, cine, coronal | T2/T1  GRE | Diaphragm nav | 10 Amplitude probability | x1.8x 4-5 mm  (333 ms) | 350x350x140 mm | 11 – 20 min (NR) | |
| Kesteren  (2019)[43] | Pancreas (1)  Stomach (1) | 2D Cartesian, interleaved, coronal | T2-w-SS | 1D nav | 10 Amplitude | 0.78×0.78x5 mm  (NR) | 400×200x55 mm | 6 min (NR) | |
| Meschini  (2019)[44] | Liver (7) | 2D Cartesian, interleaved, Oblique | T2/T1  GRE | K-medoids clustering | 8 clustering approach | 1.28×1.28 x5 mm  (180ms) | 12.5 cm slab | NR | |
| Uh  (2019)[36] | Abdominal tumours (6) | 2D Cartesian, coronal | T2w-TSE | 1D navigator | 10 Amplitude | 1.5×1.5x4 mm  (NR) | 300×300x200 mm | 4-7 min (NR) | |
| Chen  (2021)[45] | Liver (23) | 2D Cartesian, NA | T2w-TSE | 1D nav | 10 Phase | 0.94×0.94x5 mm  (NR) | NR | 5 min (NR) | |
| Meschini  (2021)[46] | Pancreas (5)  Liver (2) | 2D cartesian,  sagittal | T2/T1  GRE | NA | 8 Phase | NR | 12.5 cm slab | NR | |
| Zhang (2021)[47] | Liver (5) | 2D Cartesian,  Cine mode, axial | T2/T1  GRE | BA | 10 Phase | 1.88x1.88x5 mm  (300ms) | NA | NR | |
| Liu (2023)[48] | Liver (13) | 2D spiral, Sagittal | MRF-FISP | MRF signals | 8 Phase | 1.17x1.17x5 mm (NR) | 300x300 mm | NR | |
| *Abbreviations*: NR: not reported, second, min: minutes, MRF:MR fingerprinting, Recon: Reconstruction, MI: mutual Information, Nav: navigator. | | | | | | | | | |

| **Supplementary material E** | | | | |  |
| --- | --- | --- | --- | --- | --- |
| **Summary of the key information from 4DMRI reconstructed from multislice 2DMRI data acquisition** | | | | |  |
| **Author**  **(year)**  **[ref]** | **Validation**  **comparing to**  **4DMR** | **Validation**  **metrics** | **Other key information** | | |
|  |  |  | **Motion** | **Overall image quality of proposed 4DMRI** | |
| Liu (2014)[37] | real-time 2DMRI | MAD | 1.5 ±1.6 mm (SI), 2.1±1.9 mm (RL), and 1.1 ± 1.0 mm (AP) | NA | |
|  |  | Relative Error (%) | 14 ± 9.1% (SI), 21 ± 16% (AP), and 50 ± 35% (RL) |  |  |
| Yang  (2014)[38] | real-time 2DMRI | Tumour CC | 0.97 ± 0.03 (SI), 0.97 ± 0.02 (AP), and 0.99 ± 0.04 (RL) | (1) Superior CNR | |
|  |  | Mean Tumour Amplitude | 0.6 ± 0.2 mm (SI), 0.3 ± 0.2 mm (AP), and 0.1 4 ± 0.1 mm (RL) |  |  |
|  | 4DCT | Tumour CC | 0.95 ± 0.02 (SI), 0.94 ± 0.02 (AP), and 0.96 ± 0.02 (RL) |  |  |
|  |  | Mean Tumour Amplitude | 0.7 ± 0.0 mm (SI), 0.3 ± 0.1 mm (AP), and 0.2 ± 0.1 mm (RL) |  |  |
| Glide-hurst  (2015)[39] | NA | NA | NA | (1) High image quality with no significant artifact across all image series | |
| Paganelli  (2015)[40] | belt sorting vs  MI soring | Median RMSE | 1.4 ± 1.1 mm in phase binning  1.2 ± 0.9 mm in amplitude binning | (1) Less motion artifact | |
| Uh  (2016)[41] | real-time 2DMRI | MAD | 1.4 mm ± 0.4 mm (AP) | NA | |
| Uh  (2017)[42] | previous 4DCT studies. | peak-to-peak motion | kidney motion was <5 mm  liver and spleen motion were often >10 | NA | |
| Kesteren  (2019)[43] | Min95 binning^1^  vs Phase, Amplitude binning^1^ | NA | NA | (1) Phase and MaxIE binning both demonstrated sorting artifacts compared to MeanIE and Min95 strategies | |
| Meschini  (2019)[44] | Clustering sorting^2^ vs  MI and external surrogate (belt) | Median RMSE | 0.97-1.66 mm MI, 1.24 to 1.89 mm multiple points, 1.43 to 2.27 mm single point, 1.74 to 3.72 mm external surrogate | (1) Image quality was similar for MI and clustering sorting, but an external surrogate showed more artifacts. | |
| Uh  (2019) [36] | 4DCT | CTV to ITV volume | 2-46% 4DMRI, 9-63% 4DCT | NA | |
|  |  | DSC | 92-95% for the youngest patients with similar breathing characteristics  82-88% for other patients due to variation in breathing characteristics. |  |  |
| Chen  (2021)[45] | 4DCT | Mean Volume Differences | Statistically significant reduction in GTV (16%) and ITV (12%) | (1) Sever artifacts were found more in 4DCT compared to 4DMRI | |
|  |  | Mean GTV motion difference | 0.7 (LR), 0.9 (AP), and 1.9 (SI) mm |  |  |
| Meschini  (2021)[46] | 3D model validation with 4DMRI | DIR errors from reference points | 1.33 mm | NA | |
|  |  | Tumour COM | 1.8 mm |  |  |
| Zhang  (2021)[47] | Original 4DMRI vs TEC 4DMRI | MAD | 0.5 ± 0.5 mm (SI) diaphragm, 0.7 ± 0.4 mm (SI) tumour | (1) The CNR of the tumour showed a statistically significant increase in TEC 4DMRI | |
| Lui (2023)[48] | 4DMRF vs real-time 2DMRI | Mean amplitude motion | 1.5 ± 1.1 mm (SI) and 0.8 ± 0.6 mm (AP) | NA | |
|  |  | Mean PCC for motion | 0.95 ± 0.05 (SI) and 0.93 ± 0.09 (AP) |  |  |
| ***Abbreviations:*** BA: body area, CC: correlation coefficient, CNR: contrast-to-noise ratio, COM: tumour centers of mass, CoV: coefficient of variation, MAD: mean absolute difference, DIR: deformable image registration, DSC: Dice similarity coefficient, MAD: Mean Absolute Difference, MI: mutual information, MRF: magnetic resonance fingerprinting, mDSC: mean Dice coefficient, NA: not applicable, PCC: Pearson correlation coefficient, RMSE: diaphragm fitting root mean square errors, RL: right-left, SI: superior inferior, SSIM: structural similarity index measure, TEC: synthetic image contrast referred as tumour enhanced contrast 4DMRI .  ^1^ Min95 binning: amplitude binning where 95% of the acquired data included at end-inhale and end-exhale, MaxIE: amplitude binning, where inclusion thresholds are the end-inhale and end-exhale of diaphragm position, MeanIE: amplitude binning, where inclusion thresholds are the mean max-inhale and the mean end-exhale of diaphragm position,  ^2^ A clustering sorting: single points: the clustering is based on single internal reference point that tracked automatically, multiple points: the clustering is based on several internal reference points that tracked automatically. | | | | |  |
